# Firm Cutaneous Nodule on the Dorsal Hand: A Case Report on Fibroblastic Rheumatism

**DOI:** 10.7759/cureus.10312

**Published:** 2020-09-08

**Authors:** Reba Suri, Martin J Azzam, Michael R Heaphy

**Affiliations:** 1 Dermatology, University of Kansas Medical Center, Kansas City, USA; 2 Medicine, Southern Hills Hospital and Medical Center, Las Vegas, USA; 3 Dermatology, University of Missouri Health Care, Columbia, USA; 4 Dermatopathology, Skin Cancer and Dermatology Institute, Reno, USA

**Keywords:** arthralgia, arthritis, arthropathy, cutaneous, disease, fibroblast, fibrosing, nodule, rheumatism, rheumatology

## Abstract

Fibroblastic rheumatism (FR) is a rare dermatoarthropathy of unknown etiology. It is characterized by the onset of firm cutaneous nodules in patients with rheumatologic symptoms such as arthralgias or symmetric polyarthritis. Clinicopathologic correlation is critical in establishing the diagnosis, as the clinical manifestations can resemble other fibrosing conditions. In this report, we review the clinical and histologic features of FR, as well as conditions similar to those of the case we present, with dermatologic and rheumatologic manifestations. As part of our research, a PubMed search of the following terms was performed: "arthralgia, arthritis, arthropathy, cutaneous, disease, fibroblast, fibrosing, nodule, rheumatism, and rheumatology". This report discusses a unique case of FR diagnosed in a 37-year-old man with a single cutaneous nodule.

## Introduction

Fibroblastic rheumatism (FR) is a rare dermatoarthropathy characterized by sudden (over a period of days) onset of cutaneous nodules in patients with rheumatologic symptoms such as arthralgias, polyarthritis, fasciitis, and tendonitis [1]. The pathogenesis of FR remains to be fully elucidated. Clinicopathologic correlation and a thorough patient history are essential for a successful diagnosis of this condition. Prompt diagnosis and treatment are critical in preventing potentially detrimental joint destruction and limb contractures [1].

In this report, we describe an asymptomatic, firm, and mobile nodule on the left hand of a man who presented to a dermatology clinic. The sudden onset of this dermatologic finding, combined with a relatively extensive history of polyarthritis and arthralgias given the patient’s age, raised suspicion for a rheumatologic process. Biopsy of the cutaneous lesion showed diffuse dermal fibrosis with prominent fibroblastic proliferation. Histopathology combined with the patient’s clinical findings were most consistent with a diagnosis of FR.

## Case presentation

A previously healthy 37-year-old Caucasian man presented with a sudden onset of a single asymptomatic, firm, and mobile nodule on the left index finger, which had persisted for several months. He indicated that the lesion had appeared "overnight" and had stayed the same size. He denied any pain, pruritus, or history of trauma to the area and denied any changes to the lesion.

The patient's past medical history was notable for one year of joint pain in the right hand and bilateral knees, which he believed were work-related (he is a factory employee). He also reported bilateral plantar fasciitis and tendonitis in both wrists for which he was taking ibuprofen. Other past medical history included eczema controlled with topical steroids as needed. Pertinent family history included a 74-year-old father with diagnosed arthritis for decades.

**Figure 1:**
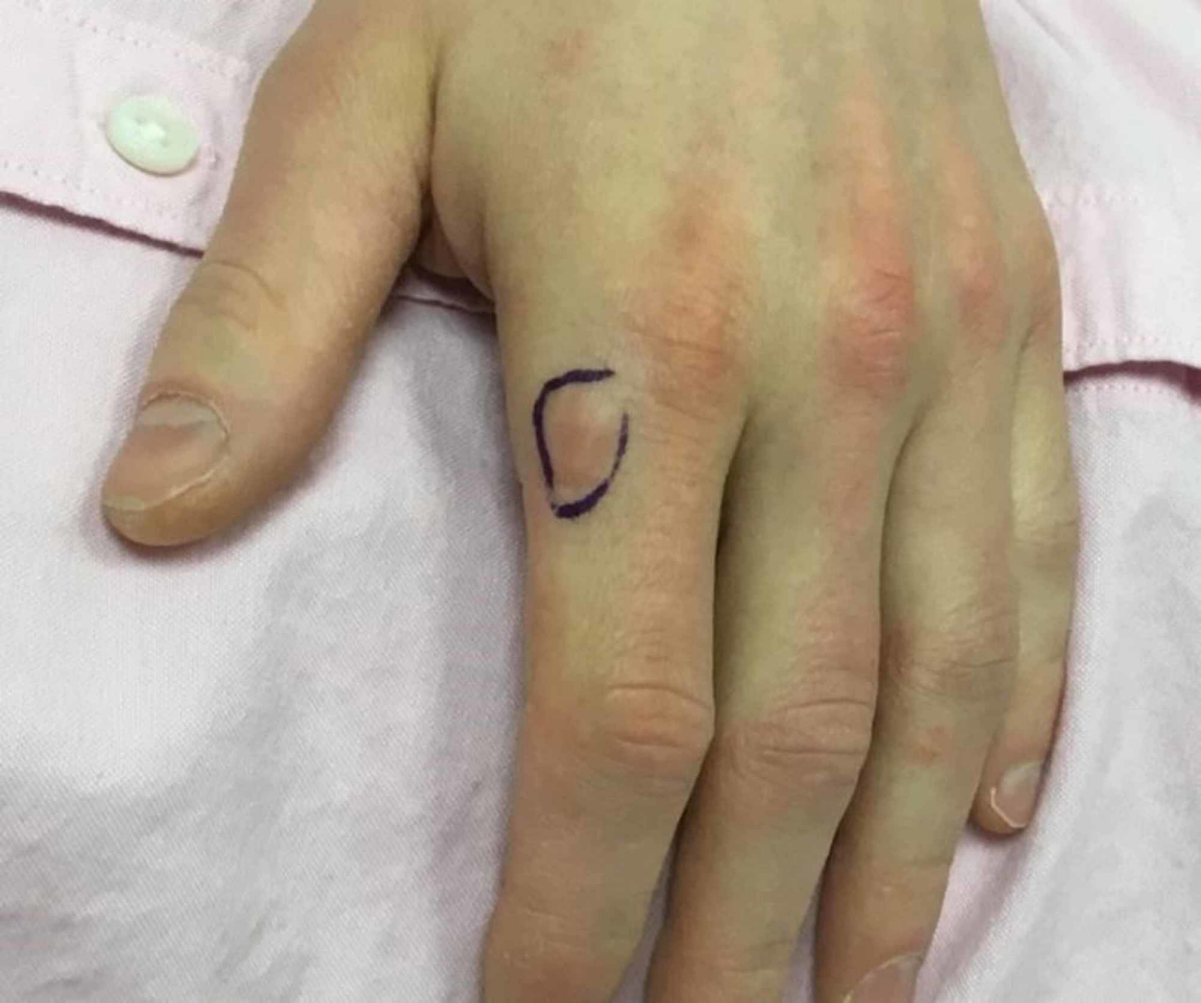
Clinical presentation of a fibroblastic rheumatism nodule on the hand of a 37-year-old Caucasian male The lesion, outlined in purple ink, consists of a 0.8 x 0.6-cm firm, flesh-colored cutaneous nodule on the index finger of the left dorsal hand

Physical exam revealed a single 0.8 x 0.6-cm indurated, flesh-colored to pink nodule on the dorsal surface of the patient’s left hand, just distal to the second metacarpophalangeal joint (Figure 1). The remainder of the patient’s physical exam and review of systems were negative.

**Figure 2:**
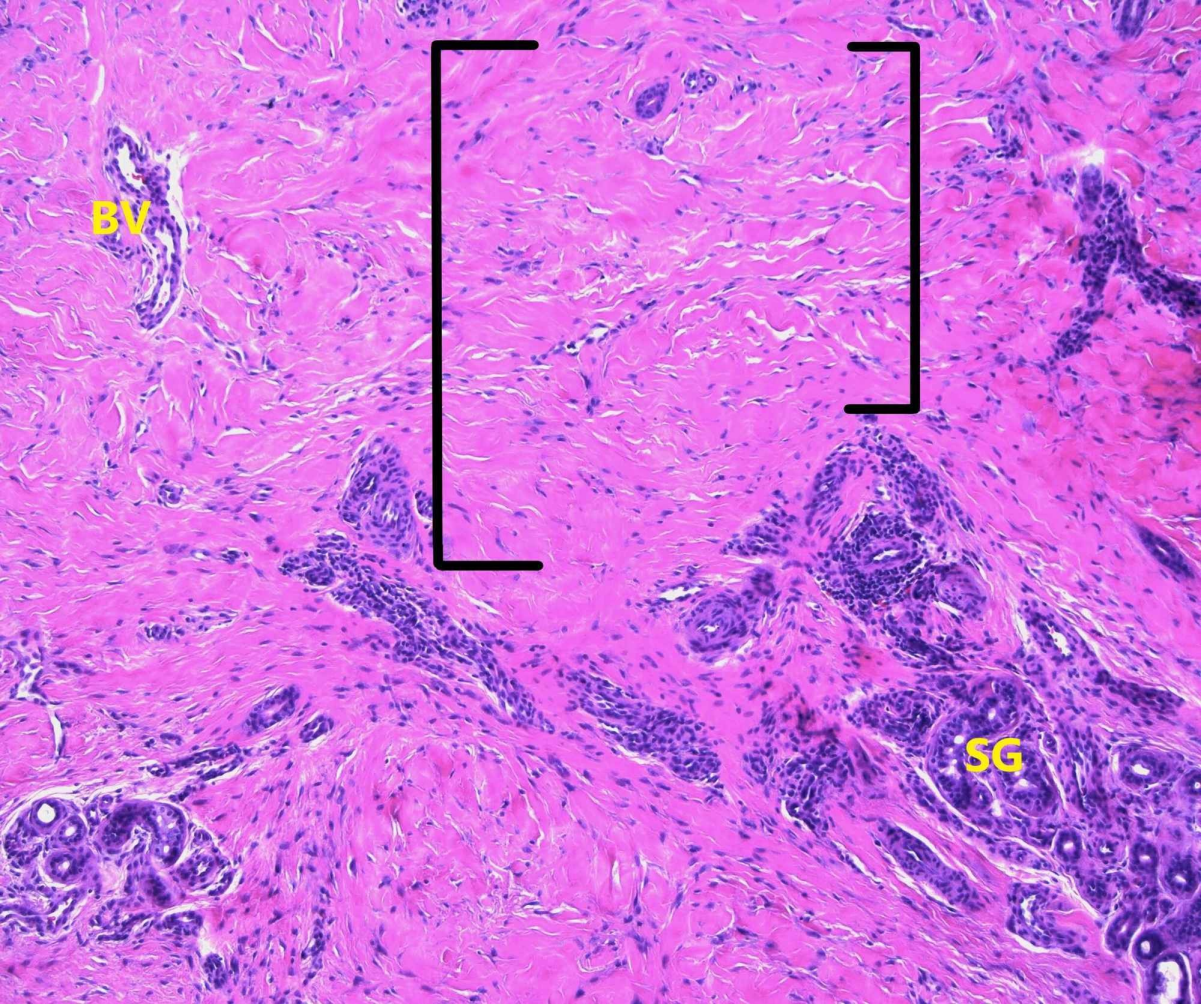
Pathology presentation of fibroblastic rheumatism nodule on the hand of a 37-year-old Caucasian male Punch biopsy of the cutaneous nodule demonstrating diffuse dermal fibrosis (dense collagen fibrils as shown between the black brackets) with prominent fibroblastic proliferation in the dermis. In addition, there is a dense concentration of sweat glands (SG) in the lower right quadrant of the high-powered field as well as a prominent blood vessel (BV) in the upper left quadrant of the high-powered field [Hematoxylin and eosin: x200]

**Figure 3:**
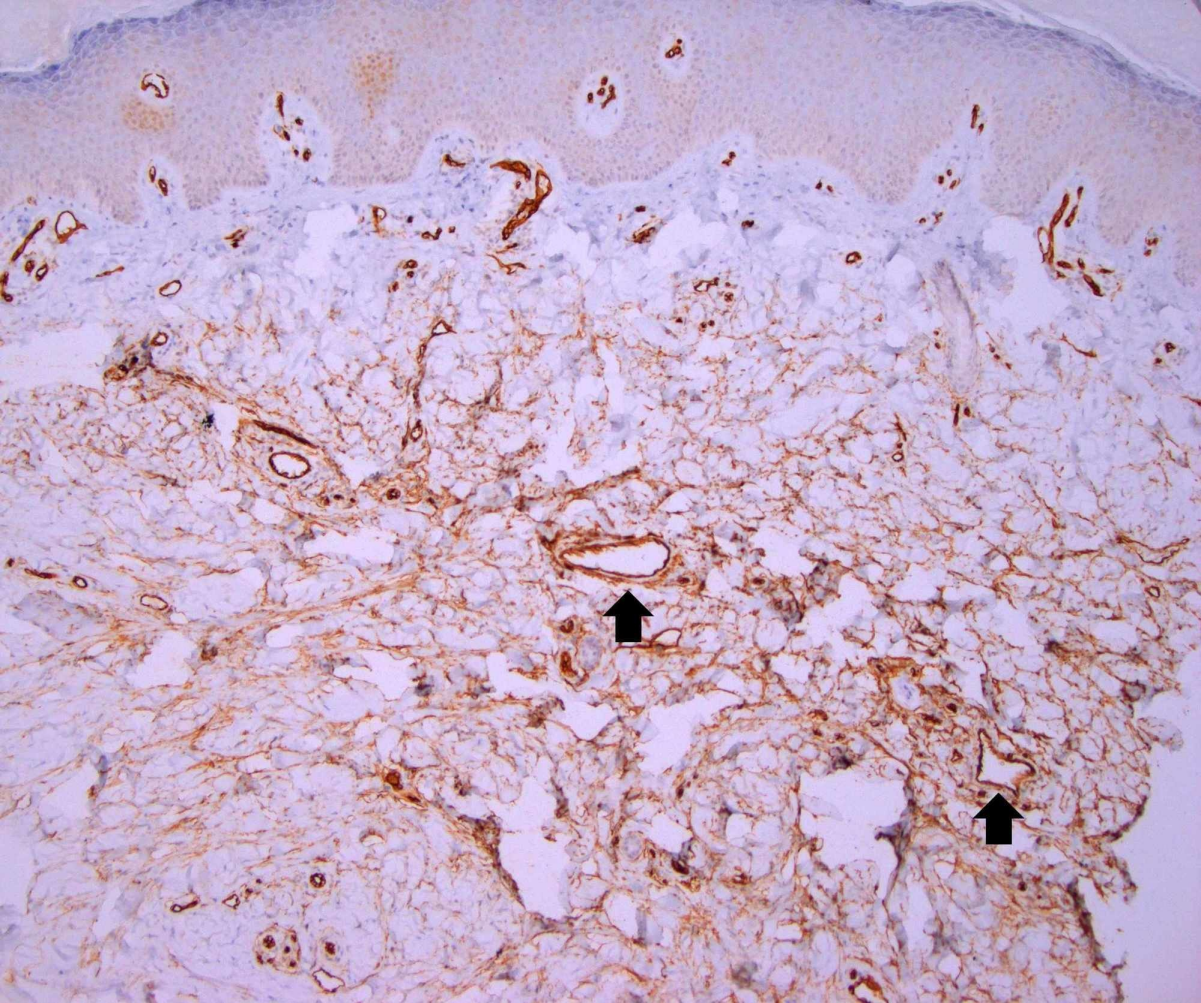
Stained pathology presentation of fibroblastic rheumatism nodule on the hand of a 37-year-old Caucasian male Punch biopsy of the cutaneous nodule showing CD34 immunohistochemical stain reacting to fibroblastic proliferation in the dermis. CD34 antigen is also seen to strongly highlight normal dermal vessels (black arrows), an expected internal control [x100]

A 3-mm punch biopsy of the lesion was performed. Histopathologic evaluation of the patient’s cutaneous nodule revealed diffuse dermal fibrosis with prominent fibroblastic proliferation (Figure 2). The spindled fibroblasts demonstrated positive immunoreactivity for CD34 (Figure 3), variable reactivity for Factor XIIIa, and Verhoeff-Van Gieson stain revealed the loss of dermal elastin fibrils. Staining for S100 and SOX10 was negative, and colloidal iron staining revealed no evidence of dermal mucin deposition. Serologic testing was negative for antinuclear antibodies and rheumatoid factor.

These pathologic findings in correlation with the clinical presentation established a diagnosis most consistent with FR. The patient was informed of the diagnosis and referred to rheumatology for further evaluation and management.

## Discussion

FR is a rare dermatoarthropathy of unknown etiology, characterized by the sudden (over a period of days) onset of firm cutaneous nodules in patients with rheumatologic symptoms such as arthralgias or symmetric polyarthritis [1]. Other clinical manifestations include sclerodactyly, thickened palmar fascia, and Raynaud’s phenomenon, though it is rare for a patient to present with all these findings simultaneously [1-3]. Cutaneous lesions in FR are typically 2-20 millimeter firm, pink to flesh-colored nodules found on the hands, ears, neck, and periarticular regions of the elbows and knees [1,4]. Histopathologic findings include an increased proliferation of dermal fibroblasts embedded within a network of dense collagen fibers, with a characteristically marked decrease or absence of elastic fibers [1]. Fibroblasts can form dense fascicles resembling scar tissue, or can alternatively be interspersed in the dermis with no organized pattern [1].

Early diagnosis and treatment of the disease are critical, as progressive involvement of the joints can lead to flexion contractures of the digits and irreversible debilitating erosive arthropathy if left untreated [1,4]. Treatment is aimed towards decreasing the abnormal proliferation of fibroblasts. Steroidal and non-steroidal anti-inflammatory agents, as well as methotrexate and interferon-alpha, have shown promising results after a few weeks to months of treatment in select patients [1].

Clinicopathologic correlation is critical in establishing the diagnosis of FR, as the clinical manifestations can resemble other fibrosing conditions [1]. Conditions on the differential that can manifest clinically with cutaneous nodules and rheumatologic symptoms include rheumatoid arthritis, nodular scleroderma, and fibromatosis (Table 1). Histopathologic findings and lab studies are thus required to exclude these conditions. Immunostains for smooth muscle actin (SMA), S100, vimentin, CD34, desmin, and epithelial membrane antigen (EMA) can be used to narrow down the differential diagnosis (Table 2) [1,5].

**Table 1:** Differential diagnosis of dermatoarthropathies that can present with cutaneous nodules

Diagnosis	Cutaneous manifestations	Rheumatologic manifestations	Histopathology
Fibroblastic rheumatism	Cutaneous periarticular nodules on the hands, sclerodactyly, Raynaud’s phenomenon	Polyarthritis (classically affecting distal joints), arthralgias, tendonitis, thickened palmar/plantar fascia	Increased proliferation of dermal fibroblasts embedded within a network of dense collagen fibers, along with the absence or decrease of elastic fibers
Rheumatoid arthritis	Firm, subcutaneous, periarticular nodules often overlying bony prominences	Symmetric polyarthritis classically affecting the metacarpophalangeal (MCP) and proximal interphalangeal (PIP) joints while sparing the distal interphalangeal (DIP) joints	Palisaded granulomas surrounding well-demarcated regions of degenerated collagen and fibrinoid necrosis in the reticular dermis and subcutaneous tissue
Nodular scleroderma	Dermal nodules on the trunk and proximal extremities, truncal sclerosis +/- Raynaud’s phenomenon, calcinosis cutis, telangiectasias	Polyarthralgias, polyarthritis, acroosteolysis, joint contractures	Dense sclerotic collagen fibers in the reticular dermis and subcutaneous tissue along with an absence of fibroblastic proliferation
Fibromatosis	Variable location depending on subtype (superficial vs. deep); indurated plaques or nodules commonly on the palms, knuckle pads, soles, or penis can be present in superficial subtypes	Diffuse thickening of palmar/plantar fascia +/- flexion contractures, osteolysis, erosive arthropathy	Fibroblasts arranged in long, cellular fascicles embedded within a dense collagenous stroma with increased vascular spaces and prominent endothelial cells; periadnexal involvement present

**Table 2:** Immunohistochemical findings in fibroblastic rheumatism

Immunohistochemical markers	Reactivity
CD34	+
Factor XIIIa	+/-
S100	-
SOX10	-
Smooth muscle actin (SMA)	+/-
Vimentin	+
Desmin	-
Epithelial membrane antigen (EMA)	-

Rheumatoid nodules typically occur in patients with a history of seropositive rheumatoid arthritis, and present as firm subcutaneous periarticular nodules often overlying bony prominences [1]. Histologic features include palisaded granulomas surrounding well-demarcated regions of degenerated collagen and fibrinoid necrosis in the reticular dermis and subcutaneous tissue [6]. The distal interphalangeal joints are spared in rheumatoid arthritis, whereas they are commonly affected in FR [4].

Cutaneous manifestations of dermal nodules, sclerodactyly, and Raynaud’s phenomenon present in scleroderma can resemble those seen in FR. Although the dense sclerotic collagen fibers in the reticular dermis and subcutaneous tissue seen in nodular scleroderma can resemble FR, nodular scleroderma can be excluded based on the absence of fibroblastic proliferation on histology [7]. Serology for anti-Scl70, anti-topoisomerase I, and anti-centromere antibodies can also be useful in differentiating between the two conditions [8].

Fibromatosis can resemble FR both clinically and histologically. Fibroblasts arranged in long, cellular fascicles embedded within a dense collagenous stroma can be seen in both conditions [1,9]. Increased vascular spaces with prominent endothelial cells, as well as periadnexal involvement seen in fibromatosis, can help differentiate it from FR [1,9].

Histopathology, combined with clinical findings of a dermal nodule, arthralgias/arthritis, and plantar fasciitis played a key role in establishing the diagnosis in our patient. A rheumatology referral was placed for further evaluation and management to prevent potentially detrimental joint destruction and limb contractures, with management primarily consisting of decreasing the abnormal proliferation of fibroblasts via the use of steroidal and non-steroidal anti-inflammatory agents [1,4]. This report delineates a unique case of FR diagnosed in a patient presenting with a single cutaneous nodule.

## Conclusions

FR is a rare dermatoarthropathy characterized by the onset of cutaneous nodules on the extremities, ears, or neck of patients with a history of rheumatologic disease. Some of the other possible clinical manifestations of this condition include sclerodactyly, thickened palmar fascia, and Raynaud's phenomenon. It is rare for a patient to present with all these findings simultaneously, and a thorough patient history including specific symptoms, the timing of symptom onset, and clinicopathologic correlation is essential for proper diagnosis. When looking at the histology of an FR nodule, increased proliferation of dermal fibroblasts embedded within a network of dense collagen fibers is fundamental to making the diagnosis. The prompt and accurate diagnosis of this potentially debilitating rheumatologic condition is of utmost importance to prevent joint destruction and limb contractures.
